# Electrophysiological characteristics of Purkinje potentials and the conduction system in premature ventricular contractions triggering ventricular fibrillation after myocardial infarction

**DOI:** 10.1093/europace/euaf249

**Published:** 2025-12-11

**Authors:** Tatsuya Hayashi, Yuki Komatsu, Shinya Kowase, Seiji Fukamizu, Koichi Nagashima, Masao Takahashi, Hitoshi Mori, Susumu Tao, Shingo Yamamoto, Yoshimi Onishi, Atsuhiko Yagishita, Jumpei Ohashi, Masato Fukunaga, Akira Mizukami, Osamu Inaba, Yuhei Kasai, Takayuki Kitai, Kennosuke Yamashita, Daigo Tokudome, Naotaka Hashiguchi, Tomofumi Nakamura, Koyo Sato, Naohiko Sahara, Kentaro Minami, Yusuke Ugata, Taku Asano, Ritsushi Kato, Tetsuo Sasano, Hideo Fujita

**Affiliations:** Division of Cardiovascular Medicine, Saitama Medical Center, Jichi Medical University, 1-847 Amanuma-cho, Omiya-ku, Saitama-si, Saitama 330-0834, Japan; Cardiovascular Division, Faculty of Medicine, University of Tsukuba, Tsukuba, Japan; Department of Heart Rhythm Management, Yokohama Rosai Hospital, Yokohama, Japan; Department of Cardiology, Tokyo Metropolitan Hiroo Hospital, Tokyo, Japan; Division of Cardiology, Department of Medicine, Nihon University School of Medicine, Tokyo, Japan; Department of Cardiology, Tokyo Metropolitan Hiroo Hospital, Tokyo, Japan; Department of Cardiology, Saitama Medical University International Medical Center, Saitama, Japan; Department of Cardiovascular Medicine, Institute of Science Tokyo, Tokyo, Japan; Division of Cardiovascular Medicine, Saitama Medical Center, Jichi Medical University, 1-847 Amanuma-cho, Omiya-ku, Saitama-si, Saitama 330-0834, Japan; Division of Cardiology, Showa University School of Medicine, Tokyo, Japan; Department of Cardiology, Tokai University School of Medicine, Isehara, Japan; Division of Cardiovascular Medicine, Saitama Medical Center, Jichi Medical University, 1-847 Amanuma-cho, Omiya-ku, Saitama-si, Saitama 330-0834, Japan; Department of Cardiology, Kokura Memorial Hospital, Kitakyushu, Japan; Department of Cardiology, Kameda Medical Center, Chiba, Japan; Department of Cardiology, Japanese Red Cross Society Saitama Hospital, Saitama, Japan; Asia Medical Group, Department of Cardiology, Sapporo CardioVascular Clinic, Sapporo Heart Center, Sapporo, Japan; Asia Medical Group, Department of Cardiology, Sapporo CardioVascular Clinic, Sapporo Heart Center, Sapporo, Japan; Department of Cardiovascular Medicine, Sendai Kosei Hospital, Miyagi, Japan; Department of Heart Rhythm Management, Yokohama Rosai Hospital, Yokohama, Japan; Department of Cardiology, Japanese Red Cross Society Narita Hospital, Chiba, Japan; Department of Cardiology, Nagoya Heart Center, Aichi, Japan; Department of Cardiology, Nagoya Heart Center, Aichi, Japan; Department of Cardiology, Saiseikai Yokohamashi Tobu Hospital, Yokohama, Japan; Department of Cardiovascular Medicine, Dokkyo Medical University, Tochigi, Japan; Division of Cardiovascular Medicine, Saitama Medical Center, Jichi Medical University, 1-847 Amanuma-cho, Omiya-ku, Saitama-si, Saitama 330-0834, Japan; Division of Cardiology, Showa University School of Medicine, Tokyo, Japan; Department of Cardiology, Saitama Medical University International Medical Center, Saitama, Japan; Department of Cardiovascular Medicine, Institute of Science Tokyo, Tokyo, Japan; Division of Cardiovascular Medicine, Saitama Medical Center, Jichi Medical University, 1-847 Amanuma-cho, Omiya-ku, Saitama-si, Saitama 330-0834, Japan

**Keywords:** Catheter ablation, Ventricular tachycardia, Purkinje, Myocardial infarction, Ventricular fibrillation

## Abstract

**Aims:**

Catheter ablation for premature ventricular contractions (PVCs) triggering ventricular fibrillation (VF) after acute myocardial infarction (AMI) has proven effective, with Purkinje potentials often serving as the target. However, the electrophysiological features of Purkinje potentials and their relationship with the conduction system in VF-triggering PVCs remain unclear.

**Methods:**

This multicentre retrospective study analysed patients who underwent catheter ablation for VF-triggering PVCs during the acute or subacute phase of AMI. Characteristics of Purkinje potentials, including retrograde conduction from the Purkinje network and subsequent anterograde conduction to the right bundle branch (RB), were evaluated for PVC morphology.

**Results:**

Fifty-three patients (mean age 66±11 years; 13% female) with 67 VF-triggering PVCs were analysed. The mean PVC width was 157 ± 42 ms, with 87% showing RB branch block morphology. Purkinje potentials preceded 72% of PVCs (mean interval 68 ± 42 ms). PVCs with preceding Purkinje potentials had narrower QRS duration (145 ± 26 ms vs. 198 ± 57 ms, *P* < 0.0001). The coupling interval from the preceding sinus beat was inversely correlated with the interval from Purkinje potential to PVC onset (*P* < 0.0001). Retrograde Purkinje conduction with subsequent anterograde RB conduction was identified in 9%. In these cases, the coupling interval from the preceding sinus beat was longer (391 ± 12 ms vs. 467 ± 34 ms, *P* = 0.041).

**Conclusion:**

Purkinje potentials show distinct properties that influence both QRS duration and PVC timing. The presence of preceding Purkinje potentials together with QRS duration may help guide ablation strategies. In rare cases, Purkinje activity conducts retrogradely from the left bundle and anterogradely through the RB, modifying PVC morphology.

## Graphical Abstract

**Graphical Abstract:**
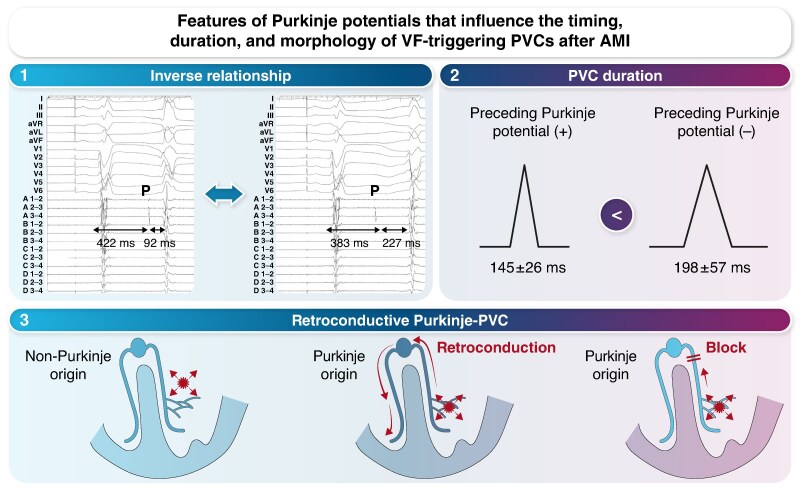
Features of Purkinje potentials that influence the timing, duration, and morphology of VF-triggering premature ventricular conductions (PVCs) after AMI are summarized as follows:The coupling interval from the preceding sinus beat was inversely correlated with the interval between the Purkinje potential and PVC onset.PVCs with preceding Purkinje potentials exhibited narrower QRS durations.Purkinje potentials were occasionally observed conducting retrogradely through the left bundle and anterogradely to the right bundle via the His bundle, thereby altering PVC morphology (‘retroconductive Purkinje PVCs’). P, Purkinje potential; AMI, acute myocardial infarction; RB, right bundle

What’s new?

- VF-triggering premature ventricular conductions (PVCs) were observed in the majority of patients with post-acute myocardial infarction VF, occurring in 90% before the procedure and 85% during the procedure.
- Preceding Purkinje potentials were identified in 72% of PVCs; these PVCs had narrower QRS durations, and shorter coupling intervals with sinus rhythm were linked to longer Purkinje-to-PVC intervals.
- A rare phenomenon (9%) was observed in which Purkinje potentials conducted retrogradely through the left bundle and orthodromically activated the RB via the His bundle, altering PVC morphology.
- Catheter ablation targeting VF-triggering PVCs was effective, with 75% of patients remaining free from VF recurrence over a mean follow-up of 195 ± 414 days, although 17% experienced appropriate implantable cardioverter-defibrillator shocks for recurrent VF.

## Introduction

Ventricular arrhythmias, including ventricular fibrillation (VF), continue to represent a refractory condition with many unresolved challenges.^1,2^ VF following an acute myocardial infarction (AMI) is a relatively common occurrence. Komatsu *et al*. demonstrated that catheter ablation targeting these triggering premature ventricular contractions (PVCs) can be an effective treatment.^3^ In many cases, VF is triggered by PVCs originating from the Purkinje network.^4,5^ However, their detailed electrophysiological characteristics, such as QRS duration and timing of appearance, remain underexplored.

Additionally, it is known that PVCs originating from Purkinje fibres may exhibit retrograde conduction from the left bundle branch to the right bundle (RB) branch through the conduction system, which could alter premature ventricular conduction (PVC) morphology.^6^ Although this phenomenon is considered rare, its exact incidence and electrophysiological features remain unknown.

In this study, we aimed to characterize the electrophysiological features of Purkinje potentials associated with VF-triggering PVCs, with particular focus on QRS duration and timing of appearance, in the acute or subacute phase following AMI. Furthermore, we assessed the incidence and electrophysiological characteristics of retrograde conduction from the left bundle branch to the RB branch via the conduction system, which may influence PVC morphology.

## Methods

### Population

We analysed patients from a multicentre, retrospective cohort who underwent catheter ablation for VF occurring during either the acute phase (within 3 days after AMI) or the subacute phase (4–30 days post-AMI). Patients were enrolled from multiple institutions. Cases prior to 2003 and patients younger than 18 years of age were excluded. Patient characteristics, including ischaemic background, 12-lead ECG findings, laboratory data, procedural details, and ablation outcomes, were evaluated.

### Mapping and ablation strategy

Ablation strategies and procedural details varied across participating centres. Radiofrequency (RF) energy was typically delivered using an irrigated catheter, with power titrated between 10 and 50 W and ablation times ranging from several seconds to a few minutes. In cases of recurrent VF, some procedures were performed under ventilator management, while others required haemodynamic support devices, such as extracorporeal membrane oxygenation (ECMO). Three-dimensional (3D) mapping systems were generally used to guide ablation, although some earlier cases were performed without this technology. When patients were in sinus rhythm and exhibited mappable PVCs, activation mapping was performed prior to ablation. If clinical PVCs had been documented before the procedure, pace mapping was additionally used to guide the ablation. When a Purkinje potential preceded a PVC, ablation targeted that specific potential. RF applications were continued until VF non-inducibility and elimination of VF-triggering PVCs were confirmed. In hemodynamically stable patients, VF induction using ventricular pacing was attempted at the discretion of the operator, with further ablation performed as needed.

### PVC and 12-lead ECG

VF-triggering PVCs were defined as follows:

1. PVCs recorded pre- or intraoperatively that induced VF in the 12-lead ECG.
2. PVCs are frequently observed in association with VF episodes, in which the morphology documented on 12-lead ECG was considered identical to that seen on monitor ECG at the onset of VF.
3. Infrequent PVCs present during VF episodes, with VF cessation following ablation.^7^

The interval from the preceding sinus rhythm QRS complex to the VF-triggering PVC, as well as the interval from the preceding Purkinje potential to the PVC, was measured. The timing of the sinus rhythm, QRS complex, and the PVC was determined based on the onset of the QRS complex on the 12-lead electrocardiogram. For intracardiac electrogram analysis, the presence of His bundle or RB potentials preceding PVCs was assessed to identify retrograde conduction from Purkinje potentials to the His bundle, often originating from Purkinje firing in the left ventricular septum. A diagnosis of ‘retroconductive Purkinje PVC’ was made when a left ventricular Purkinje excitation preceded the PVC, and an RB potential—similar to that observed during sinus rhythm—was seen preceding the local ventricular activation recorded by the right ventricular catheter.

### Statistical analysis

All values were expressed as medians with interquartile ranges. Continuous variables were compared using one-way ANOVA. Categorical variables expressed as numbers and percentages between different groups were compared with Pearson’s *χ*^2^ test, and two-group comparisons were performed with Fisher’s exact test. Statistical significance was defined as *P* < 0.05. JMP® Pro 17.2 (SAS Institute Inc., Cary, NC, USA) was used for analysis.

## Results

### Patient characteristics

Between December 2003 and December 2023, 59 patients were enrolled from 17 regional hospitals in Japan. Of these, 6 patients were excluded due to unavailable ECG data on VF-triggering PVCs, leaving 53 patients for analysis. The average age was 66 ± 11 years, with seven females (13%). Mean ejection fraction (EF) and left ventricular end-diastolic dimension (LVDd) were 32 ± 10% and 56 ± 8 mm, respectively. Preoperative amiodarone was administered in 50 cases (94%). Catheter ablation was performed during the subacute phase of AMI in 47 patients (89%) and in the acute phase in 6 patients (11%). Haemodynamic support was provided prior to ablation in 31 cases (58%). Of the total cohort, 25 patients (48%) had ST-segment elevation myocardial infarction (STEMI), and pre-ablation percutaneous coronary intervention (PCI) was performed in 43 cases (81%), involving single-vessel lesions in 8 cases (15%), two-vessel lesions in 24 cases (45%), and three-vessel lesions in 21 cases (40%). Staged PCI was performed in 10 (19%) patients. Among these, chronic total occlusion (CTO) lesions were identified in 14 patients (26%). Of these patients with CTO lesions, 5 out of 14 (36%) had lesions that corresponded to the VF-triggering PVC origin (*Table 1*).

**Table 1:** Clinical characteristics

	*n* = 53
Age (year)	66 ± 11
Female (%)	7 (13%)
Structural heart disease	0 (0%)
HT (%)	34 (64%)
DM (%)	27 (51%)
AF (%)	16 (30%)
CKD (eGFR < 60) (%)	29 (55%)
CKD on HD	2 (4%)
COPD (%)	1 (2%)
Stroke (%)	5 (9%)
EF (%)	32 ± 10
LVDd (mm)	56 ± 8
*NYHA class (before VF occurrin*g)	
I	13 (25%)
II	12 (23%)
III	9 (17%)
IV	19 (36%)
Overdrive pacing for VF suppression (before ABL) (%)	19 (36%)
Haemodynamic support device (before ABL) (%)	31 (58%)
Use of sedation and respirator (before ABL) (%)	46 (87%)
Amiodarone (%)	50 (90%)
Β-blocker (%)	49 (93%)
Class I drug (%)	15 (28%)
*Infarcted area (ant or inf or multi)*	
Ant (%)	18 (34%)
Inf (%)	1 (2%)
Multi (%)	34 (64%)
STEMI (%)	25 (48%)
*Acute or subacute (72 h–30 days)*	
Acute	6 (11%)
Subacute	47 (89%)
*Number of occluded/stenosis coronary artery branches*	
1	8 (15%)
2	24 (45%)
3	21 (40%)
*Presence of CTO*	14 (26%)
PCI (before ABL) (%)	43 (81%)
CABG (before ABL) (%)	14 (26%)
ICD (before ABL) (%)	8 (15%)

DM, diabetes Mellitus; HT, hypertension; AF, atrial fibrillation; CKD, chronic kidney disease; COPD, chronic obstructive pulmonary disease; VF, ventricular fibrillation; ABL, ablation; CTO, chronic total occlusion; PCI, percutaneous coronary intervention; CABG, coronary artery bypass graft; LVDd, left ventricular diastolic diameter; ICD, implantable cardioverter-defibrillator; NYHA, New York Heart Association.

### ECG and triggering PVC morphology　

**Figure 1:**
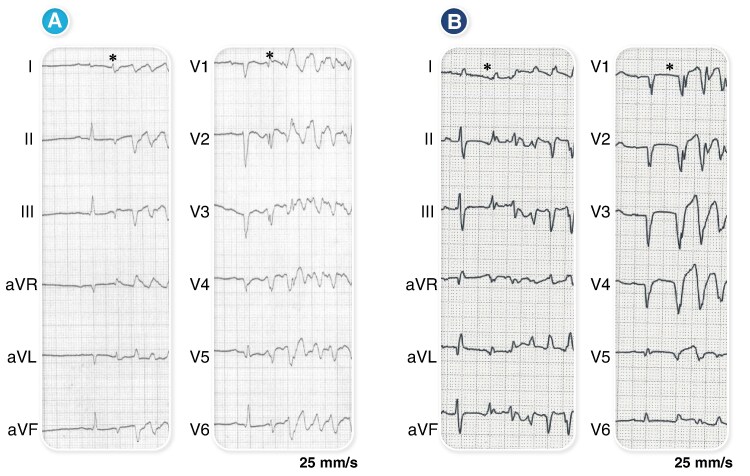
VF-triggering PVC with RBBB-like morphology (*A*) and LBBB-like morphology (*B*) are displayed. ^＊^VF-triggering PVC. RBBB, right bundle branch block; LBBB, left bundle branch block.

Triggering PVCs were identified in 47 patients (90%) preoperatively, with 28 patients (54%) presenting multiple morphologies. During the procedure, 45 patients (85%) had VF-triggering PVCs, with 23 patients (43%) exhibiting multiple morphologies (average 1.2 ± 0.7). Non-sustained ventricular tachycardia (NSVT) or VF occurred in 33 patients (62%) during the procedure, with 38 individuals (72%) experiencing VF following triggering PVCs. In total, 67 PVCs that met the inclusion criteria were identified as VF-triggering PVCs. The mean PVC width was 157 ± 42 ms. Among these 67 PVCs, 58 (87%) exhibited right bundle branch block (RBBB)-like morphology, while 9 (13%) displayed left bundle branch block (LBBB)-like morphology (*Figure 1*). Regarding axis deviation, 32 PVCs (48%) demonstrated a superior axis, and 35 PVCs (52%) exhibited an inferior axis (*Table 2*).

**Table 2:** PVC chacarteristics

PVC	(*n* = 67)
PVC morphology recorded (before ABL)	47 (90%)
Multiple PVC morphology (before ABL)	28 (54%)
Appearance of Trigger PVC during ABL	45 (85%)
Multiple PVC during procedure	23 (43%)
ISP or burst pacing for PVC induction	14 (26%)
Appearance of VF/VT during procedure	33 (62%)
VF/VT following triggering PVC	38 (72%)
PVC width (ms)	157 ± 42
Coupling interval between preceding sinus rhythm and PVC (ms)	397 ± 86
RBBB-like morphology/LBBB-like morphology	58 (87%)/9 (13%)
superior axis/inferior axis	32 (48%)/35 (52%)

VF, ventricular fibrillation; VT, ventricular tachycardia; ISP, isoproterenol; ABL, ablation; RBBB, right bundle branch block; LBBB, left bundle branch block.

### Purkinje potential during ablation

**Figure 2:**
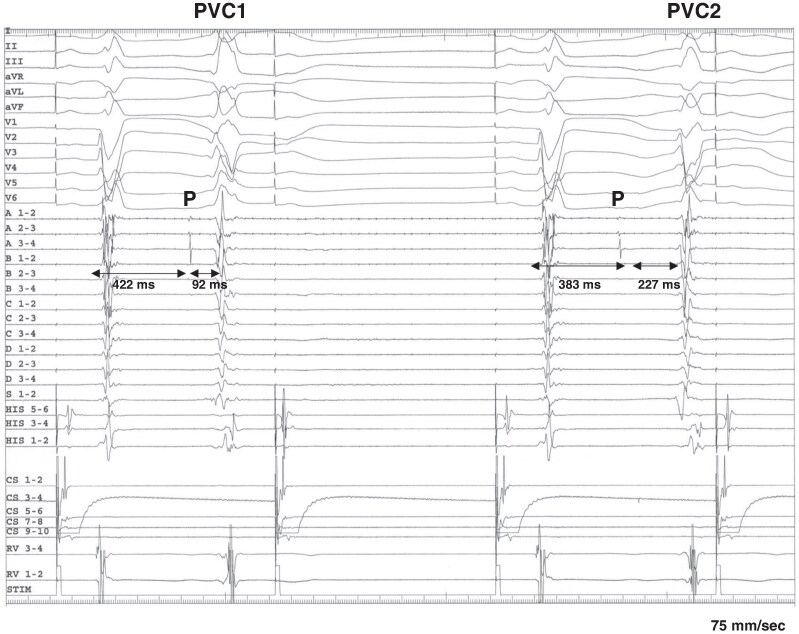
Preceding Purkinje potentials before the PVCs are evident in this case. During atrial pacing from the coronary sinus (CS), PVC1 exhibited a longer coupling interval from the preceding sinus QRS to the Purkinje potential, but a shorter interval from the Purkinje potential to PVC onset compared to PVC2. Notably, although the Purkinje potentials were similar between these two PVCs, the QRS morphologies differed. P, Purkinje potential; CS, coronary sinus; RV, right ventricle; A–D represents each spline of the HD grid.

**Figure 3:**
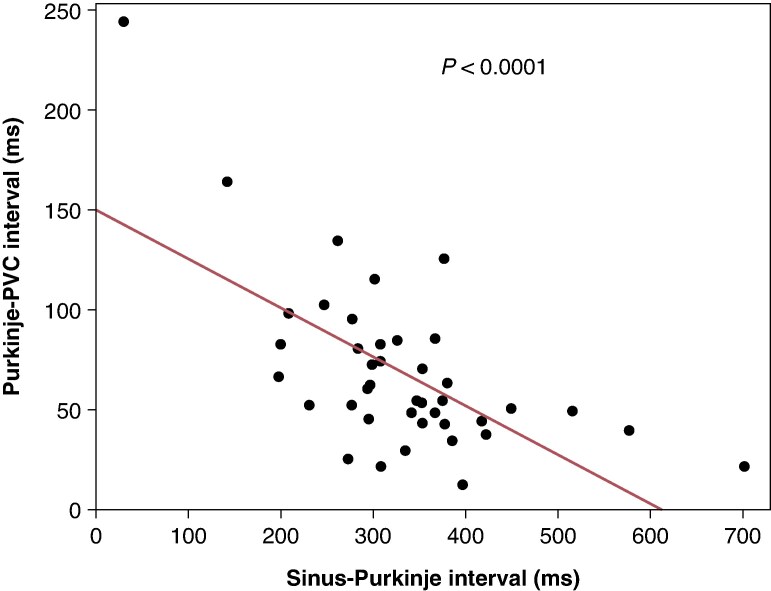
The precedence of the Purkinje potential to the PVC has been shown to inversely correlate with the interval between the Purkinje potential and the preceding sinus rhythm.

**Figure 4:**
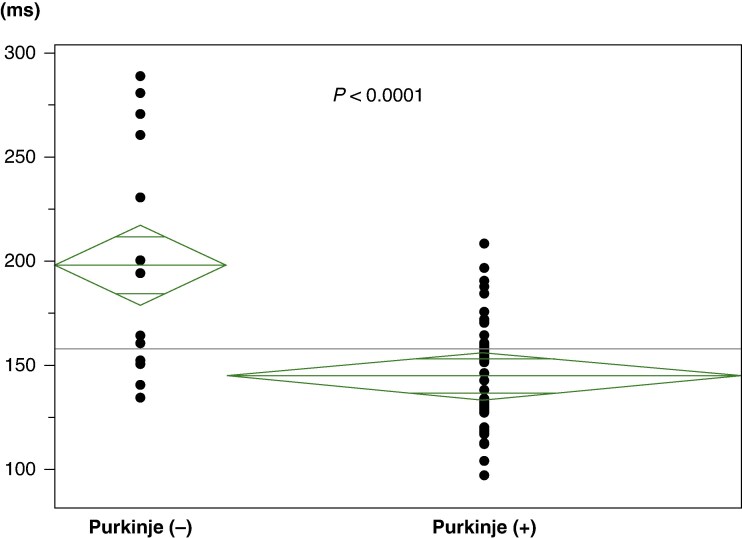
PVCs with preceding Purkinje potentials had a narrower QRS duration compared to PVCs without preceding Purkinje potentials.

Among the 67 PVCs analysed, 48 (72%) exhibited preceding Purkinje potentials, occurring on average 68 ± 42 ms before the PVCs (*Table 3*). The average coupling interval from the preceding sinus rhythm QRS to the PVC was 399 ± 86 ms [of these, eight (12%) cases were assessed during atrial pacing]. Notably, a shorter coupling interval between Purkinje potentials and sinus rhythm correlated with a longer interval from Purkinje potentials to PVC onset (*P* < 0.001) (*Figures 2* and *3*). When comparing PVCs with preceding Purkinje potentials to those without, PVCs with preceding Purkinje potentials exhibited a narrower QRS duration than those without (145 ± 26 ms vs. 198 ± 57 ms, *P* < 0.0001) (*Figure 4*). There were no significant differences in the presence of a preceding Purkinje potential when comparing PVCs with LBBB-like morphology to those with RBBB-like morphology [6/9 (67%) vs. 40/55 (73%), *P* = 0.70]. There was no significant difference in the detection rates of Purkinje potentials among the mapping systems [CARTO 26/38 (68%) vs. EnSite 18/24 (75%) vs. none 4/5 (80%), *P* = 0.78] or among the different catheter types [multipolar catheter 26/35 (74%) vs. decapolar catheter 5/9 (56%) vs. ablation catheter 17/23 (74%), *P* = 0.52].

**Table 3:** PVC and preceding Purkinje potential

	PVC (*n* = 67)
Presence of preceding Purkinje potential	48 (72%)
Precedence of Purkinje potential to PVC (ms)	68 ± 42
Coupling interval from preceding sinus rhythm to PVC (ms)	399 ± 86
Preceding His bundle or right bundle potential to PVC (%)	6 (9%)
Interval between sinus rhythm to Purkinje (ms)	340 ± 99

**Figure 5:**
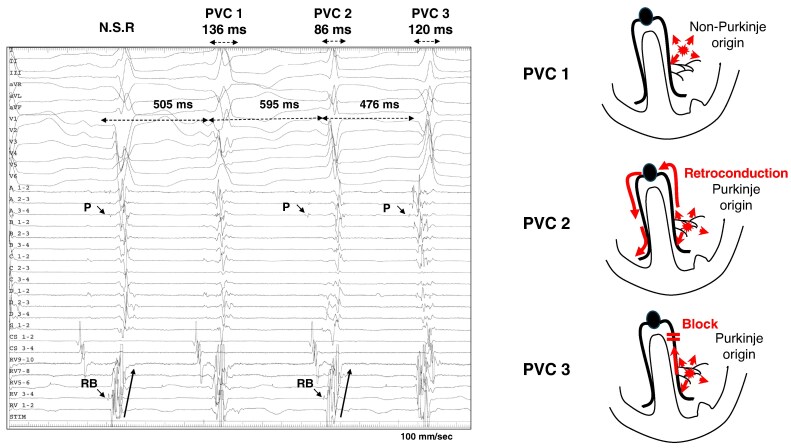
During normal sinus rhythm, a preceding Purkinje potential and a right bundle (RB) potential can be observed on the HD grid spline a. However, for PVC1, no preceding Purkinje potential is identified, and the RB potential is also not apparent. In contrast, PVC2 shows a distinct preceding Purkinje potential on the HD grid spline A, and an RB potential preceding the PVC is evident on the RV lead. This RB potential exhibits the same polarity as the RB potential during normal sinus rhythm. These findings suggest that excitation originating from the left ventricular Purkinje fibres conducts retrogradely to the His bundle and anterogradely to the RB, influencing the morphology of the PVC. On the other hand, PVC3 shows a preceding Purkinje potential but lacks a preceding RB potential, indicating that retrograde conduction from the left bundle is blocked. It is notable that, compared to PVC3, PVC2 has a longer coupling interval from the preceding QRS complex (595 ms vs. 476 ms) and a narrower QRS duration (86 ms vs. 120 ms). NSR, normal sinus rhythm; P, Purkinje potential; RB, right bundle potential; CS, coronary sinus; RV, right ventricle; A–D represents each spline of the HD grid.

Among the 46 cases where preceding Purkinje potentials were observed in PVCs, six PVCs (9%) in 3 patients exhibited preceding RB potentials. This finding suggests that excitation from the Purkinje fibres has been conducted retrogradely from the left bundle to the RB orthodromically through the His bundle (retroconductive Purkinje PVC) (*Figure 5*). Of the six retroconductive Purkinje PVCs, three exhibited a RBBB-like morphology with an inferior axis, two had a RBBB-like morphology with a superior axis, and one showed a LBBB-like morphology with an inferior axis. Retroconductive Purkinje PVC had longer coupling intervals from the preceding sinus QRS to PVC compared to those other PVCs without RB potentials (391 ± 12 ms vs. 467 ± 34 ms, *P* = 0.041) (*Table 4*). Retroconductive Purkinje PVC tended to have a narrower QRS duration compared to those without, although the difference was not statistically significant (133 ± 18 ms vs. 161 ± 6 ms, *P* = 0.12) (see Supplementary material online, *Figure S1*).

**Table 4:** Comparison of PVC between PVC with and without RB potential

	RB potential (+), *n* = 6	RB potential (−), *n* = 61	*P*-value
RBBB morphology	5 (83%)	53 (87%)	1.0000
Presence of initial Q (q)	4 (67%0	48 (79%)	0.61
PVC width (ms)	133 ± 18	161 ± 6	0.12
Preceding sinus rhythm to Purkinje (ms)	404 ± 40	330 ± 16	0.093
Purkinje to PVC (ms)	69 ± 7	63 ± 17	0.76
Interval from preceding sinus rhythm to PVC (ms)	467 ± 34	391 ± 12	0.041

RB, right bundle.

Regarding the recording of RB potentials, EnSite appeared to record them more frequently [CARTO 0/38 (0%) vs. EnSite 6/24 (25%) vs. none 0/5 (0%), *P* = 0.0027]. However, because RB potentials were recorded from the right ventricular catheter, this finding was considered to reflect institutional differences in procedural methods rather than differences between mapping systems. Comparison of the clinical and electrophysiological characteristics between STEMI and non-STEMI (NSTEMI) patients revealed some differences in clinical features but no significant differences in electrophysiological findings (*Table 5*). The key features of Purkinje potentials that influence the timing, duration, and morphology of VF-triggering PVCs after AMI are summarized in the *Graphical Abstract*.

**Table 5:** Comparison of STEMI vs. NSTEMI

	STEMI (*n* = 25)	NSTEMI (*n* = 28)	*P*-value
*Baseline characteristics*			
Age (year)	65 ± 11	66 ± 11	0.77
Female (%)	23 (92%)	23 (82%)	0.43
Structural heart disease	0 (0%)	0 (0%)	1.00
HT (%)	15 (44%)	19 (68%)	0.58
DM (%)	15 (60%)	12 (43%)	0.27
AF (%)	3 (12%)	13 (46%)	0.0079
CKD (eGFR < 60) (%)	13 (52%)	16 (57%	0.79
CKD on HD			
COPD (%)	0 (0%)	1 (4%)	1.00
Stroke (%)	1 (4%)	4 (14%)	0.35
EF (%)	31 ± 10	33 ± 11	0.41
LVDd (mm)	53 ± 7	59 ± 8	0.0041
NYHA class (before VF occurring)			
I	8 (32%)	5 (18%)	
II	5 (20%)	7 (25%)	0.51
III	5 (20%)	4 (14%)	
IV	7 (28%)	12 (43%)	
Overdrive pacing for VF suppression (before ABL) (%)	6 (24%)	13 (46%)	0.15
Haemodynamic support device (before ABL) (%)	19 (76%)	12 (43%)	0.025
Use of sedation and respirator (before ABL) (%)	23 (92%)	23 (82%)	0.43
Amiodarone (%)	24 (96%)	26 (93%)	1.0
β-blocker (%)	21 (84%	28 (100%)	0.043
Class I drug (%)	5 (20%)	10 (36%)	0.24
*Infarcted area (ant or inf or multi)*			
Ant (%)	13 (52%)	5 (18%)	
Inf (%)	0 (0%)	1 (4%)	0.025
Multi (%)	12 (48%)	22 (79%)	
Acute or subacute (72 h–30 days)			
Acute	24 (96%)	23 (82%)	
Subacute	1 (4%)	5 (18%)	0.20
Number of occluded/stenosis coronary artery branches			
1	5 (20%)	3 (11%)	
2	15 (60%)	9 (32%)	0.022
3	5 (20%)	16 (57%)	
Presence of CTO	9 (36%)	5 (18%)	0.21
PCI (before ABL) (%)	25 (100%)	18 (64%)	0.0008
CABG (before ABL) (%)	3 (12%)	11 (39%)	0.032
ICD (before ABL) (%)	2 (8%)	6 (21%)	0.26
*EPS charactarristics*			
Multiple PVC morphology (before ABL)	12/25 (48%)	11/28 (39%)	0.59
ISP or burst pacing for PVC induction	4/25 (16%)	10/28 (36%)	0.13
ISP or burst pacing for PVC induction	14/25 (56%)	19/28 (68%)	0.41
PVC width (ms)	156 ± 36	157 ± 38	0.91
Coupling interval from preceding sinus rhythm to PVC (ms)	418 ± 94	384 ± 70	0.16
Presence of preceding Purkinje potential	16/25 (47%)	21/28 (75%)	0.55
Precedence of Purkinje potential to PVC (ms)	63 ± 25	70 ± 62	0.67
Interval between sinus rhythm to Purkinje (ms)	330 ± 123	328 ± 84	0.95

DM, diabetes mellitus; HT, hypertension; AF, atrial fibrillation; CKD, chronic kidney disease; COPD, chronic obstructive pulmonary disease; VF, ventricular fibrillation; ABL, ablation; CTO, chronic total occlusion; PCI, percutaneous coronary intervention; CABG, coronary artery bypass graft; LVDd, left ventricular diastolic diameter; ICD, implantable cardioverter-defibrillator; EPS, electrophysiological study.

### Ablation outcome and follow-up

During catheter ablation, haemodynamic support was utilized in 29 (55%) cases, while sedation with mechanical ventilation was employed in 48 (91%) (*Table 6*). Ablation was targeted at Purkinje potentials in 49 (92%) of the patients, performed during both PVCs and sinus rhythm. The most common successful ablation sites were located in the left ventricular septum (40 cases, 77%), followed by the left ventricular anterior wall (26 cases, 48%), inferior wall (5 cases, 9%), and lateral wall (4 cases, 8%). Additional sites included the posterior papillary muscle (three cases), anterior papillary muscle (one case), epicardium (one case), and right ventricle (one case). The relationship between the infarcted area and the ablation area is summarized in Supplementary material online, *Table S1*. After the ablation, PCI was undertaken in five (9%) patients. Of these, PCI was performed in none (0%) of the patients with single-vessel disease, three (13%) with two-vessel disease, and two (10%) with three-vessel disease.

**Table 6:** Ablation procedure and follow-up

	*n* = 53
Haemodynamic support device (during ABL) (%)	26 (49%)
Use of sedation and respirator (during ABL) (%)	48 (91%)
*3D mapping system*	
CARTO	32 (60%)
EnSite	16 (31%)
none	5 (9%)
*Ablation site*	
LV anterior (%)	26 (48%)
LV septal (%)	40 (77%)
LV lateral (%)	4 (8%)
LV inferior (%)	5 (9%)
Others (%)	6 (11%)
Use of ISP or burst pacing to introduce PVC	14 (26%)
Ablation targeting the Purkinje potential (including the Purkinje potential during sinus rhythm)	49 (92%)
Disappearance of PVC (%)	47 (89%)
*VT/VF inducible by stimulation after ABL (%)*	
Inducible	7 (13%)
Non-inducible	37 (70%)
Induction not performed	9 (17%)
*Number of total session*	1.2 ± 0.5
1	41 (77%)
2	11 (21%)
3	1 (2%)
Recurrence of VF after final session (%)	13 (25%)
ICD implantation after ABL (%)	31 (58%)
Death during hospitalization (%)	12 (23%)

PVC, premature ventricular conduction; VT, ventricular tachycardia; VF, ventricular fibrillation; ABL, ablation; ISP, isoproterenol

The average number of procedures, including repeat ablations for recurrent cases, was 1.2 ± 0.5. Following the final ablation, 37 (70%) cases became non-inducible for VF. There was no significant difference in VF recurrence between patients with and without preceding Purkinje potentials before PVCs [30/37 (81%) vs. 10/16 (63%), *P* = 0.18]. In addition, there was no significant association between VF recurrence and QRS duration (153 ± 36 ms vs. 165 ± 39 ms, *P* = 0.34), and no other parameters were identified that were associated with ablation success (see Supplementary material online, *Table S2*).

Implantable cardioverter-defibrillator (ICD) implantation was performed in 31 (58%) patients. The average follow-up period was 1247 ± 1606 days. After the final ablation, 37 cases (70%) became non-inducible for VF, the average VF-free survival period was 195 ± 414 days, and 9 (17%) patients had appropriate ICD shock for VF recurrence. In-hospital mortality post-ablation occurred in 12 (23%), with causes detailed in Supplementary material online, *Table S3*. After the final ablation sessions, 75% of patients remained free from VF recurrence.

## Discussion

### Major findings

The main findings of this study are as follows:

- VF-triggering PVCs were recorded in 90% of patients pre-procedure and 85% during the procedure in cases of VF following AMI.
- Purkinje potentials preceding PVCs were detected in 72% of cases, and PVCs with preceding Purkinje potentials exhibited a narrower QRS duration compared to those without. Notably, a shorter coupling interval between Purkinje potentials and preceding sinus rhythm was associated with a longer interval from Purkinje potentials to PVC onset.
- In rare instances (9%), retrograde conduction of Purkinje potentials could activate the RB orthodromically through the His bundle, leading to changes in PVC morphology.
- Catheter ablation targeting VF-triggering PVCs yielded favourable results, with 75% of patients remaining free from VF recurrence during a follow-up of 195 ± 414 days, while 9 patients (17%) experienced appropriate ICD shocks for recurrent VF.

### Preceding Purkinje potential

VF-triggering PVCs after AMI often arise from excitation by Purkinje potentials located at the periphery of the conduction system.^8,9^ Catheter ablation targeting these PVCs has proven effective in suppressing VF. During such procedures, Purkinje potentials that precede the PVCs are frequently observed and serve as the primary targets for ablation.^10^ These Purkinje potentials typically originate from the infarcted area adjacent to the His–Purkinje system, particularly within the border zone of the infarcted region.^11^ In the current study, preceding Purkinje potentials were detected in 48 of the 67 PVCs analysed (72%). In contrast, Komatsu *et al*.^12^ reported the presence of Purkinje potentials in 90% of cases during both sinus rhythm and PVCs, which is higher than the findings in our study. This discrepancy may be attributed to the multicentre nature of our research, where not all centres may have extensive experience with the procedure, leading to variations in ablation techniques. Also, we observed that when comparing PVCs with preceding Purkinje potentials to those without, PVCs with preceding Purkinje potentials exhibited a narrower QRS duration than those without. It is possible that Purkinje potentials do not always precede PVCs, not only because recording Purkinje activity can be technically challenging but also because some PVCs may originate directly from the left ventricular myocardium and serve as the trigger for VF. Such PVCs, which are not preceded by Purkinje activity within the conduction system, may therefore exhibit a relatively wider QRS duration. These considerations suggest that, if the target VF-triggering PVC is relatively narrow (mean 145 ± 26 ms), an ablation strategy focusing on identifying preceding Purkinje potentials could be considered. This is also consistent with the concept of directly targeting the origin of PVCs. Conversely, if the target PVC is relatively wide, the likelihood of a preceding Purkinje potential being absent is higher, and ablation should be guided by the site of earliest ventricular activation of the PVC.

Furthermore, we observed an inverse relationship between the coupling interval from the Purkinje potential to the preceding sinus QRS complex and the interval from the Purkinje potential to PVC onset: the shorter the coupling interval to the sinus QRS, the longer the interval to PVC onset. A previous case report similarly described this inverse relationship between the coupling intervals of Purkinje potentials to the preceding and following local ventricular potentials during PVCs.^10^ To our knowledge, the present study is the first to demonstrate this finding in a multicentre analysis involving a large number of cases. The underlying mechanism of this delay may involve decremental conduction properties at the junction between the Purkinje network and the infarcted myocardium or conduction delay within the diseased Purkinje system itself. Previous studies have reported that decremental conduction may occur within the Purkinje system, particularly in association with diseased Purkinje potentials, which could explain, at least in part, the inverse relationship observed in our study between the coupling interval of Purkinje potentials to sinus rhythm and the Purkinje-to-QRS interval.^13^ On the other hand, similar findings have been demonstrated in reports of ablation guided by local abnormal ventricular activities (LAVA) (in which 80% of the patients had ischaemic heart disease) as well as in studies of ‘DEEP’ mapping, where early extrastimulation was used to define ablation targets (in which 100% of the patients had ischaemic heart disease).^14,15^ In these cases, myocardial damage due to ischaemia was shown to cause pacing-dependent intramyocardial conduction delay. As a result, even early stimulation originating from the Purkinje system can lead to a prolongation of conduction time to the ventricle. In the present study, we demonstrated the inverse relationship between the coupling interval of Purkinje potentials to sinus rhythm and the Purkinje-to-QRS interval; however, we cannot definitively determine the underlying mechanism. It is conceivable that either mechanism, or both, may contribute to the observed conduction delay. Further data are needed to clarify the precise mechanisms underlying the delay from Purkinje potential to PVC onset.

Occasionally, a long delay can be observed between the Purkinje potential and the onset of the PVC.^16^ In such cases, the site is thought to be more distant, making the ablation procedure more complex. Therefore, ablation targeting the Purkinje potential, as the presumed origin of the PVC, may represent a more efficient strategy. Indeed, in our study, Purkinje potentials preceding PVCs were identified in 72% of cases, suggesting that ablation at these sites may provide a more fundamental treatment strategy. This concept is also consistent with the notion that when targeting the PVC breakout site, PVCs may persist by shifting to another exit site. Conversely, in cases without identifiable Purkinje potentials (28% in this study), ablation was directed to the PVC breakout site. In addition, when PVCs occurred only rarely, pace mapping served as an important adjunctive tool to guide therapy.

### Retroconduction from Purkinje to the conduction system

In VF-triggering PVCs associated with Purkinje pre-excitation, retrograde conduction from the Purkinje potential in the left ventricle to the His bundle can occur, resulting in orthodromic RB potentials preceding the surface PVCs recorded.^6^ This phenomenon has also been observed during left bundle area pacing.^17^

In our study, we identified such occurrences of ‘retroconductive Purkinje PVC’ in six PVCs, marking the first report to demonstrate that retrograde conduction occurs spontaneously in PVCs, while also addressing its underlying mechanisms and incidence. The low incidence rate might be due to limited awareness and insufficient RB potential recording in retrospective studies.

Retroconductive Purkinje PVCs demonstrated longer coupling intervals from the preceding sinus rhythm QRS complex to PVC onset than other VF-triggering PVCs. This extended interval likely provides additional time for PVC depolarization to propagate retrogradely from Purkinje fibres in the left ventricle to the RB.

Theoretically, retroconductive Purkinje PVC may exhibit narrower QRS widths than other PVCs because they utilize RB conduction to activate the right ventricle. However, in this study, there was no significant difference in PVC width between retroconductive Purkinje PVCs and others (133 ± 18 ms vs. 161 ± 6 ms, *P* = 0.12). This lack of significance may result from the low sensitivity for detecting RB potentials in this retrospective analysis, potentially leading to the inclusion of some retroconductive Purkinje PVCs with narrow widths in the comparison group. In the present study, PVCs preceded by Purkinje potentials were narrower than those without preceding Purkinje potentials. One possible explanation is that a portion of the conduction system may participate in intraventricular propagation. Another possibility, although less frequently detected, is that retroconductive Purkinje PVCs may have contributed to the narrower QRS morphology. Of the nine VF-triggering PVCs with LBBB-like morphology, six (67%) exhibited a preceding Purkinje potential. The presence of LBBB-like morphology despite the Purkinje potential originating in the LV may also be explained by the presence of retroconductive Purkinje PVC. There was no significant difference in the presence of a preceding Purkinje potential when comparing PVCs with LBBB-like morphology to those with RBBB-like morphology in this study. However, given that retroconductive Purkinje PVCs have the property of conducting anterogradely through the RB, and considering the possibility that such PVCs may have been underestimated, the proportion of LBBB-like morphology in cases with preceding Purkinje potentials may also have been underestimated.

Despite its rarity, recognizing these occurrences is essential, as retrograde RB conduction can significantly influence PVC morphology. It is therefore crucial to record both Purkinje and RB potentials during ablation of VF-triggered PVCs to accurately assess the underlying mechanisms of the PVCs.

### Limitation

Although this study is multicentre and includes all patients with VF-triggering PVCs from 2003 across 17 centres, including territorial hospitals, the number of PVCs that induce VF remains relatively small, limiting the robustness of our analysis.

We included not only pre- or intraoperative PVCs that were directly documented as inducing VF but also those that frequently occurred when VF was present or less frequent, but VF disappeared after ablation. This broader inclusion criterion was employed to capture and analyse a more significant number of PVCs that may induce VF, which could have reduced specificity.

Additionally, as this was a retrospective, multicentre study, recording His or RB potentials during ablation—particularly at the time of PVC occurrence—was not mandatory and, at some centres, was technically difficult. This limitation may have contributed to underestimating the presence of His or RB potentials due to retrograde conduction.

## Conclusions

Purkinje potentials preceding VF-triggering PVCs exhibit unique electrophysiological characteristics, including a tendency towards narrower PVC width and an inverse relationship between the coupling interval from the preceding sinus rhythm and the interval to PVC onset. These findings may have important implications for ablation strategies. In rare cases, Purkinje activity during VF-triggering PVCs has been reported to conduct retrogradely through the left bundle branch and subsequently antegradely through the RB branch, thereby altering the PVC morphology.

## Supplementary Material

euaf249_Supplementary_Data

## Data Availability

The data supporting the findings of this study are available upon request to the corresponding author, T.H.
