# Supplementary material for: Electrophysiological characteristics of Purkinje potentials and the conduction system in premature ventricular contractions triggering ventricular fibrillation after myocardial infarction
Source: Europace. 2025 Dec 11;27(12):euaf249. doi: 10.1093/europace/euaf249 (PMC12696382; doi:10.1093/europace/euaf249)
Supplement: euaf249_Supplementary_Data [file euaf249_supplementary_data.zip › TableS1.docx]

| ***ABL location*** | | | | | | | | | |
| --- | --- | --- | --- | --- | --- | --- | --- | --- | --- |
|  |  | ant  (N=26) | sep  (N=40) | inf  (N=5) | lateral  (N=4) | PPM  (N=3) | APM  (N=1) | Epi  (N=1) | RV  (N=1) |
| ***Infarcted area*** | ant | 11 (61%) | 14 (78%) | 0 (0%) | 2 (11%) | 1 (33%) | 1 (100%) | 0 (0%) | 0 (0%) |
|  | inf | 0 (0%) | 0 (0%) | 0 (0%) | 0 (0%) | 0 (0%) | 0 (0%) | 0 (0%) | 1(100%) |
|  | multi | 15 (44％) | 26 (76%) | 4 (12%) | 2 (6%) | 2 (67%) | 0 (0%) | 1(100%) | 0 (0%) |

**Table S1**
