# Supplementary material for: Electrophysiological characteristics of Purkinje potentials and the conduction system in premature ventricular contractions triggering ventricular fibrillation after myocardial infarction
Source: Europace. 2025 Dec 11;27(12):euaf249. doi: 10.1093/europace/euaf249 (PMC12696382; doi:10.1093/europace/euaf249)
Supplement: euaf249_Supplementary_Data [file euaf249_supplementary_data.zip › TableS3.docx]

|  | N=12 |
| --- | --- |
| Intestinal disease | 3 |
| Sepsis | 1 |
| Heart Failure | 2 |
| VT/VF | 2 |
| Multiple Organ Failure | 1 |
| Unknown | 3 |

**Table S3: All causes of mortality**
