# Supplementary material for: Electrophysiological characteristics of Purkinje potentials and the conduction system in premature ventricular contractions triggering ventricular fibrillation after myocardial infarction
Source: Europace. 2025 Dec 11;27(12):euaf249. doi: 10.1093/europace/euaf249 (PMC12696382; doi:10.1093/europace/euaf249)
Supplement: euaf249_Supplementary_Data [file euaf249_supplementary_data.zip › TablesS2.docx]

|  | **No VT/VF recurrence**  **After final session**  **(N=40)** | **VT/VF recurrence**  **After final session**  **(N=13)** | **P value** |
| --- | --- | --- | --- |
| PVC width | 153±7 | 165±10 | 0.34 |
| Interval from preceding sinus rhythm to PVC | 390±14 | 409±23 | 0.50 |
| Interval from Purkinje to PVC | 69±9 | 57±17 | 0.54 |
| Interval from preceding Sinus rhythm to Purkinje | 320±23 | 328±41 | 0.86 |
| Sinus rhythm width | 107±5 | 122±8 | 0.093 |
| CRBBB morphology | 35 (88%) | 12 (92%) | 1.00 |
| Presence of Q (q) in lead V1 | 32 (80%) | 10 (77%) | 1.00 |
| Presence of Q in lead V1 | 13 (33%) | 3 (23%) | 0.73 |
| Presence of q in lead V1 | 19 (48%) | 7 (54%) | 0.76 |
| Hemodynamic support device (during ABL) (%) | 20 (50%) | 6 (46%) | 1.00 |
| Use of sedation and respirator (during ABL) (%) | 36 (90%) | 12 (92%) | 1.00 |
| ***3D mapping system*** |  |  |  |
| CARTO | 23 (58%) | 9 (69%) |  |
| EnSite | 12 (30%) | 4 (31%) | 0.40 |
| none | 5 (13%) | 0 (0%) |  |
| Appearance of Trigger PVC during PVC | 35 (88%) | 10 (77%) | 0.39 |
| Multiple PVC during PVC | 17 (43%) | 6 (46%) | 1.00 |
| Ablation site |  |  |  |
| LV anterior (%) | 19 (48%) | 7 (54%) | 0.69 |
| LV septal (%) | 32 (80%) | 8 962%) | 0.27 |
| LV lateral (%) | 3 (8%) | 1 (8%) | 1.00 |
| LV inferior (%) | 2 (5%) | 2 (17%) | 0.22 |
| Others (%) | 3 (8%) | 3 (23%) | 0.15 |
| Ablation targeting the Purkinje potential (including Purkinje potential during Sinus rhythm) (%) | 37 (93%) | 12 (92%) | 1.00 |
| PVC during procedure | 35 (88%) | 10 (77%)( | 0.86 |
| Multiple during PVC | 17 (43%) | 6 (46%) | 1.00 |
| VT/VF following PVC | 24 （60%） | 9 (69%)) | 0.74 |
| Use of ISP to introduce PVC/burst pacing | 8 (20%) | 6 (46%) | 0.080 |
| NSVT/VF during procedure | 24 (60%) | 9 (69%) | 0.74 |
| Presence of Purkinje potential | 30 (75%) | 7 (54%) | 0.18 |
| Ablation to Purkinje potential (including Purkinje potential during S.R ) | 37 (93%) | 12 (92%) | 1.00 |
| Disappearance of PVC (%) | 37 (93%) | 10 (77%) | 0.15 |
| ***VT/VF inducible by stimulation after ABL (%)*** |  |  |  |
| inducible | 3 (8%) | 4 (31%) |  |
| No inducible | 31 (77%) | 6 (46%) | 0.056 |
| Induction not performed | 6 (15%) | 3 (23%) |  |
| ***Number of Total Session*** |  |  |  |
| 1 | 30 (75%) | 11 (84%) |  |
| 2 | 10 (25%) | 1 (8%) | 0.10 |
| 3 | 0 (0%) | 1 (8%) |  |

**Table S2**

**Comparisons of the PVC and ablation data between patients with and without VF recurrence after the final procedure**
