# Supplementary figures and images for: Electrophysiological characteristics of Purkinje potentials and the conduction system in premature ventricular contractions triggering ventricular fibrillation after myocardial infarction
Source: Europace. 2025 Dec 11;27(12):euaf249. doi: 10.1093/europace/euaf249 (PMC12696382; doi:10.1093/europace/euaf249)

**Figure S1**

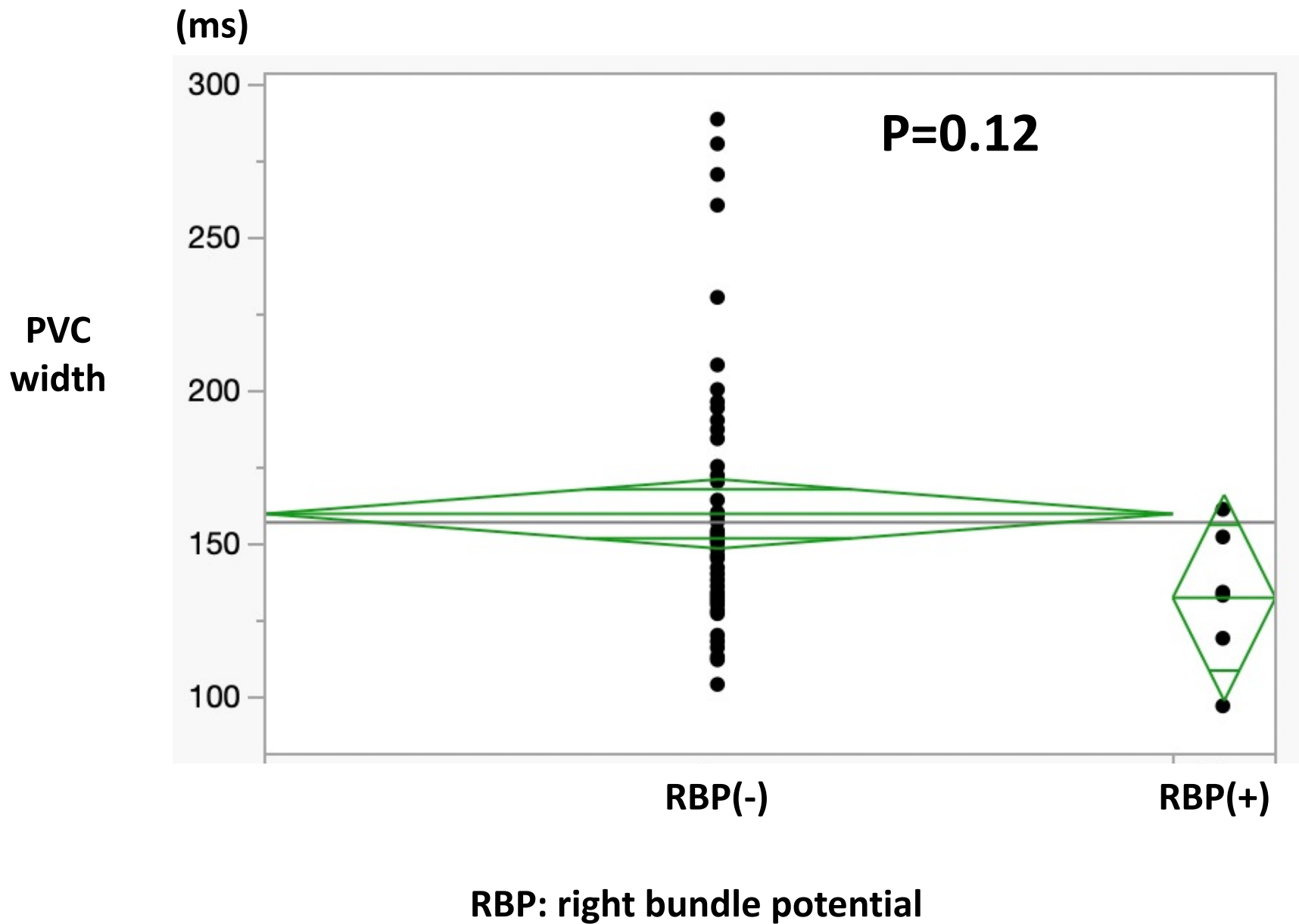

Supplement: euaf249_Supplementary_Data [file euaf249_supplementary_data.zip › FigureS1.pdf]
